# Epithelial Alarmins in Serum and Exhaled Breath in Patients with Idiopathic Pulmonary Fibrosis: A Prospective One-Year Follow-Up Cohort Study

**DOI:** 10.3390/jcm8101590

**Published:** 2019-10-02

**Authors:** Sebastian Majewski, Karolina Szewczyk, Adam J. Białas, Joanna Miłkowska-Dymanowska, Paweł Górski, Wojciech J. Piotrowski

**Affiliations:** Department of Pneumology and Allergy, Medical University of Lodz, 90-153 Lodz, Poland; karolina.szewczyk@umed.lodz.pl (K.S.); adam.bialas@umed.lodz.pl (A.J.B.); joanna.milkowska-dymanowska@umed.lodz.pl (J.M.-D.); pawel.gorski@umed.lodz.pl (P.G.); wojciech.piotrowski@umed.lodz.pl (W.J.P.)

**Keywords:** epithelial-derived cytokines, epithelial alarmins, IL-25, IL-33, TSLP, exhaled breath, EBC, idiopathic pulmonary fibrosis, IPF, fibrotic lung disease

## Abstract

Background: Recently, epithelial alarmins have been shown to play important roles in non-allergen driven respiratory diseases like idiopathic pulmonary fibrosis (IPF). Little is known about the expression of the epithelial alarmins in IPF. Methods: This study aimed to prospectively examine interleukin (IL)-25, IL-33, and thymic stromal lymphopoietin (TSLP) levels in the serum and exhaled breath condensate (EBC) in patients with IPF before and after one-year of antifibrotic treatment. A total of 82 volunteers, including 52 patients diagnosed with IPF that qualified for antifibrotic therapy as well as 30 controls, were examined. All study participants underwent baseline peripheral blood and EBC sampling. In 35 out of 52 IPF subjects, a follow-up sampling was performed after 12 months of antifibrotic treatment. Concentrations of alarmins in the serum and EBC were evaluated by means of ELISA. Results: Baseline TSLP concentrations were significantly elevated in patients with IPF compared to controls both in the serum (*p* < 0.05) and EBC (*p* < 0.0001). Baseline IL-25 and IL-33 serum and EBC levels did not differ significantly between IPF subjects and controls. Prospective analysis of changes in the epithelial alarmin levels showed significantly decreased IL-25 and TSLP EBC concentrations after 12 months of antifibrotic treatment (*p* < 0.05), which was observed in the subgroup of IPF patients treated with pirfenidone, but not in those treated with nintedanib. In stable patients with IPF over a study period (absolute forced vital capacity (FVC) % of predicted decline/year ≤ 5%, *n* = 25), a significant decrease in the EBC levels of both IL-25 and TSLP after 12 months of antifibrotic treatment was noted (*p* < 0.05), whereas, in progressor IPF patients (absolute FVC % of predicted decline/year > 5%, *n* = 10), a significant decrease was noted in the IL-25 EBC levels only (*p* < 0.05). Conclusions: Elevated TSLP levels in patients with IPF and their significant decrease in the lung compartment during antifibrotic therapy in stable patients with IPF, but not in progressors, support its significant contribution to pro-fibrotic type 2 immune responses in IPF. Noted changes in the epithelial alarmins concentration in the lung compartment during pirfenidone therapy may suggest its possible interaction with epithelial alarmins pathways in IPF.

## 1. Introduction

Airway and alveolar epithelium constitute the first line of interaction with an atmospheric environment. This continuous interaction between the epithelium and inhaled environment is crucial in the pathobiology of diverse respiratory conditions. Consequences of damage to the respiratory epithelium or dysregulated activation of it caused by exposure to the atmospheric insults, allergens, cigarette smoke, or microbiological pathogens may play central roles in the immune response, chronic airway inflammation, remodeling, and lung fibrosis [1].

Several studies have identified an important role of airway epithelial-derived cytokines, recognized as epithelial alarmins, including interleukin (IL)-25, IL-33, and thymic stromal lymphopoietin (TSLP) in the pathogenesis of allergic asthma [1,2,3]. Alarmins are released from the epithelium and other local stromal compartments when cells are damaged or stressed by allergens, pollutants, or pathogens and thereby trigger the production of the type 2 cytokines including IL-5, IL-9, and IL-13 by diverse effector cells of the innate and adaptive immune systems [4,5]. However, respective roles and the importance of epithelial alarmins in patients with non-allergen driven chronic respiratory conditions are underrecognized.

Idiopathic pulmonary fibrosis (IPF) is dominated by profibrotic non-allergen type 2 immunity, and recently, epithelial alarmins have been suggested to play a significant role in the disease pathogenesis [6,7,8,9,10]. Chronic and repetitive injuries to the alveolar epithelial cells are considered crucial events for initiation of the pathogenic process in IPF. The injured epithelium is a source of proinflammatory and profibrotic factors that orchestrate the wound healing process in the lung [11,12]. Once the alveolar epithelium is injured, the epithelial and fibroblastic pathways of wound healing become activated. Accumulating evidence suggests that epithelial alarmins may be involved in the profibrotic responses in IPF. It has been demonstrated recently, that the upregulated IL-25 axis contributes to lung fibrosis by promoting profibrotic phenotype changes of the alveolar epithelial cells and direct activation of fibroblasts [10]. Several studies have highlighted the importance of IL-33 in the enhancement of proinflammatory and profibrotic injury induced by various insults in IPF [6,7,13,14]. It has also been suggested that TSLP may promote the recruitment of immune and inflammatory cells crucial to wound repair to sites of injury and that fibroblasts represent a cellular source and target of TSLP [15]. Only a few clinical studies evaluated epithelial alarmins in the cross-sectional analyses in the different interstitial lung diseases (ILD), including IPF, hypersensitivity pneumonitis (HP), non-specific interstitial pneumonia (NSIP), sarcoidosis, systemic sclerosis-associated ILD (SSc-ILD), and rheumatoid arthritis-associated ILD (RA-ILD) [8,16,17,18,19,20]. The results of these studies offer compelling evidence supporting the contribution of epithelial alarmins to the perpetuation of pulmonary fibrosis. In this context, it is reasonable to study epithelial alarmins expression and their roles in the pathobiology of IPF. However, studies prospectively evaluating IL-25, IL-33, and TSLP concentrations in systemic and lung compartments in patients with IPF are lacking.

Although measurements of the epithelial alarmins directly in the lung would be ideal, IPF patients are often very sick and debilitated, and local lung samples are extremely hard to obtain. Exhaled breath condensate (EBC) is a non-invasive approach for sampling airway epithelial lining fluid and offers a convenient tool to provide biomarkers of inflammation. Measurements of such markers are increasingly being explored for studying airway inflammation qualitatively and quantitatively for research purposes and potential clinical applications [21,22]. Previous studies showed that increased levels of epithelial alarmins are detectable in the EBC of patients with asthma and COPD [23,24,25]. More recently, the feasibility of epithelial alarmin measurements in the EBC has also been shown in patients with IPF [26].

In the present study, we, therefore, examined IL-25, IL-33, and TSLP levels in the serum and EBC of patients with IPF and analyzed their relationships with clinical characteristics. Moreover, we analyzed changes in the epithelial alarmin levels before and after 12 months of antifibrotic treatment.

## 2. Experimental Section

### 2.1. Study Population

In this study, a total of 82 volunteers, including 52 patients diagnosed with IPF that qualified for antifibrotic treatment as well as 30 controls, were examined. IPF was diagnosed according to the international guidelines and recommendations [27]. Control subjects were not burdened by any significant comorbidity and had no previous lung disease history or symptoms. The study protocol was reviewed and approved by the Ethics Committee of the Medical University of Lodz (approval number RNN/66/17/KE, date 14.03.2017), and the study was performed according to the Declaration of Helsinki principles. All participants gave written informed consent before the start of any study procedures.

### 2.2. Methods

All study participants underwent clinical assessments, including medical history, physical examination, and spirometry. In addition, for IPF subjects, the extended pulmonary function assessment included the single-breath transfer factor of the lung for carbon monoxide (T_LCO_), and a functional assessment using the six-minute walk test (6MWT) was performed. Peripheral blood samples and EBC were collected from all study participants baseline and in 35 out of 52 IPF subjects after 12 months of antifibrotic therapy. IPF subjects available for a second study timepoint at the 12-month follow-up visit were also assessed using spirometry, T_LCO_, and 6MWT. Concentrations of epithelial alarmins were evaluated by enzyme-linked immunosorbent assay (ELISA).

### 2.3. Pulmonary Function Assessments

Spirometry and the single-breath T_LCO_ measurements were performed using the Lungtest 1000 system (MES, Cracow, Poland) according to ATS/ERS standards [28,29]. Forced expiratory volume in 1 second (FEV_1_), forced vital capacity (FVC), FEV_1_/FVC%, and T_LCO_ corrected for hemoglobin concentration were recorded. For the expression of pulmonary function results as a % of predicted values, we used the Global Lung Function Initiative (GLI) reference values [30,31].

### 2.4. The Six-Minute Walk Test (6MWT)

The 6MWT is used for the evaluation of functional exercise capacity in patients with different chronic respiratory conditions. In the present study, 6MWT was performed using the methodology specified by the Polish Respiratory Society guidelines [32]. Briefly, all IPF subjects were instructed to walk as far as possible for 6 minutes. The 6MWT was performed in a flat, covered corridor which was 30 meters long and meter–by–meter marked. When the test was finished, the distance covered was calculated.

### 2.5. Composite Physiologic Index (CPI)

CPI was developed as a tool to reflect the morphologic extent of fibrosis in patients with IPF on high-resolution computed tomography (HRCT) [33]. It is calculated based on the lung function parameters as follows:

91.0−(0.65 × T_LCO_ percentage of the predicted value (% pred))−(0.53 × FVC% pred) + (0.34 ×  FEV_1_% pred)

CPI has been shown to provide prognostic information in patients with IPF.

### 2.6. Blood Samples Processing

Peripheral venous blood samples were collected into serum separator tubes (SST) (BD Dickinson). Samples were left for 30 minutes at room temperature to allow the blood to clot and then centrifuged for 15 minutes at 1000× *g*. Serum samples were stored at −80 °C until further processing.

### 2.7. Exhaled Breath Condensate (EBC)

The EBC was collected using a commercially available condenser (Thermo Haake EK20, EcoScreen, Erich Jaeger GmbH, Hoechberg, Germany) according to the recommendations specified by the European Respiratory Society [34]. Briefly, all subjects were asked to breath out spontaneously for 10 minutes through a mouthpiece equipped with a saliva trap. The respiratory rate during the EBC collection ranged from 15 to 20 breaths per minute. All study subjects wore a nose clip and rinsed their mouths with distilled water before and in the seventh minute of EBC collection to reduce nasal contamination of the sample. Collected condensate was immediately frozen in −80 °C until ELISA measurements.

### 2.8. Alarmin Levels Measurements

Peripheral blood and EBC concentrations of IL-25, IL-33, and TSLP were measured in duplicate using commercially available enzyme-linked immunosorbent assays (R&D Systems, Inc., MN, USA) according to the manufacturer’s instructions. The assays minimum detectable dose (MDD) for human IL-25 ranged from 11.5 pg/mL to 750 pg/mL, for IL-33 ranged from 0.069 pg/mL to 1.51 pg/mL, and for TSLP ranged from 1.05 pg/mL to 9.87 pg/mL. In the case of values lower than the method sensitivity limit, the samples were quantified based on the extrapolation of standard curves generated for each set of samples assayed.

### 2.9. Statistical Analysis

Data were analyzed using Dell Statistica (version 13.1, Dell Inc, Tulsa, OK, USA). Normality of data distribution was tested with the Shapiro–Wilk test. Continuous data are expressed as mean with standard deviation (SD) for normally distributed data or as median with interquartile range (IQR) for nonparametric data. Categorical data are presented as absolute numbers and relative frequencies. Data were analyzed using the Mann-Whitney *U* test, paired *t*-test, or Wilcoxon signed-rank test, depending on data normality and variance homogeneity. Correlations were analyzed with the Spearman correlation coefficient, according to the assumptions of the tests. The significance was accepted at *p* < 0.05. The results were evaluated with the program GraphPad Prism 8 (GraphPad Software, La Jolla, San Diego, CA, USA).

## 3. Results

### 3.1. Study Participants

The summary of characteristics of study participants is shown in Table 1. IPF patients were generally older and had greater smoking exposure and worse lung function than control subjects.

### 3.2. Baseline Epithelial Alarmin Levels in the Serum

**Figure 1:**
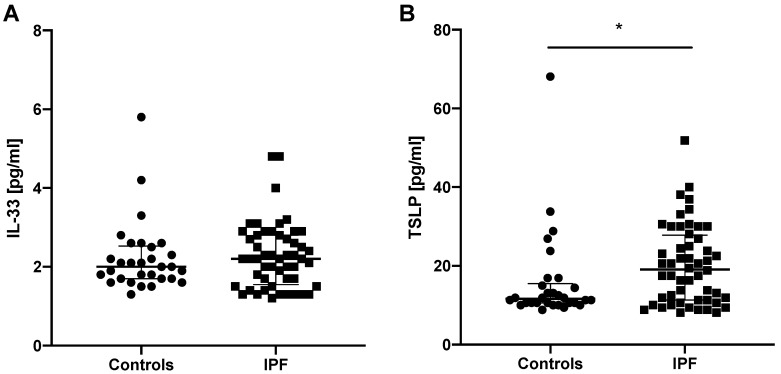
Baseline epithelial alarmin levels in the serum of controls and patients with IPF. Notes: Panels showing: (**A**) baseline IL-33 serum levels; (**B**) baseline TSLP serum levels; * *p* < 0.05. IL-25—interleukin 25, IL-33—interleukin 33, TSLP—thymic stromal lymphopoietin, IPF—idiopathic pulmonary fibrosis.

IL-25 serum levels were measurable only in 6 out of 82 subjects studied; therefore, this precluded further statistical analysis of IL-25 serum levels in our study. IL-33 and TSLP levels were measurable in all subjects studied. No significant difference was noted in IL-33 serum levels in IPF subjects (2.20 (1.60–2.80) pg/mL) compared to controls (2.00 (1.70–2.50) pg/mL). We noted significantly elevated TSLP serum levels in patients with IPF (19.10 (11.30–27.50) pg/mL) compared to controls (11.60 (10.60–15.00) pg/mL; *p* < 0.05), see Figure 1.

### 3.3. Baseline Epithelial Alarmin Levels in the EBC

**Figure 2:**
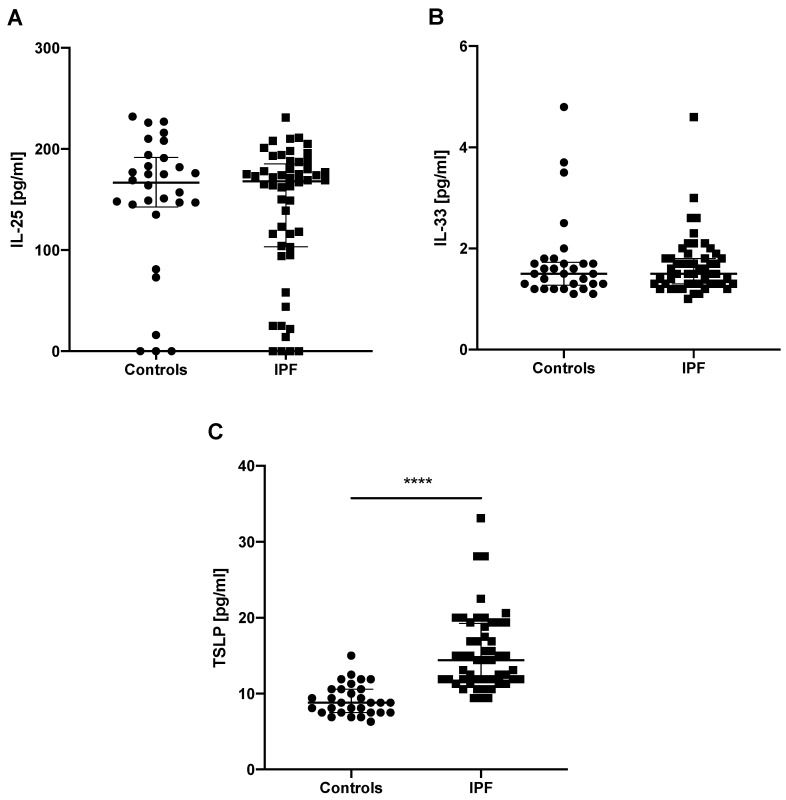
Baseline epithelial alarmin levels in the EBC of controls and patients with IPF. Notes: Panels showing: (**A**) baseline IL-25 EBC levels; (**B**) baseline IL-33 EBC levels; (**C**) baseline TSLP EBC levels; **** *p* < 0.0001. EBC—exhaled breath condensate, IPF—idiopathic pulmonary fibrosis, IL-25—interleukin 25, IL-33—interleukin 33, TSLP—thymic stromal lymphopoietin.

IL-25 EBC levels were measurable in 75 out of 82 subjects studied, whereas IL-33 and TSLP EBC levels were measurable in all study subjects. No significant difference was noted for IL-25 EBC levels in IPF subjects (168.00 (103.50–183.50) pg/mL) compared to controls (166.50 (145.00–191.00) pg/mL). Similarly, IL-33 EBC levels were not statistically different between IPF subjects (1.50 (1.30–1.80) pg/mL) and control subjects. (1.50 (1.30–1.70) pg/mL). TSLP EBC levels were significantly elevated in patients with IPF compared to controls (14.40 (11.90–19.90) pg/mL vs. 8.80 (7.50–10.60) pg/mL; *p* < 0.0001), see Figure 2.

### 3.4. Associations of Epithelial Alarmin Levels in the Serum and EBC of Patients with IPF

**Figure 3:**
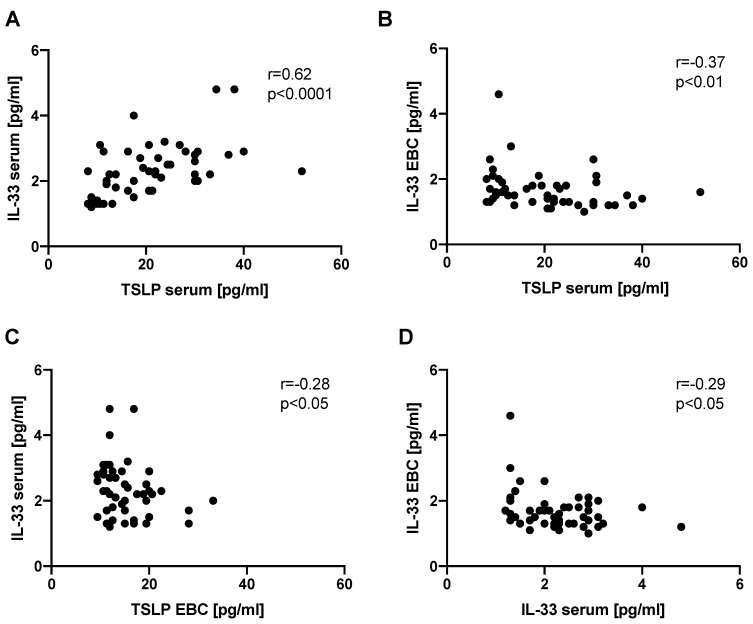
Graphical presentation of significant correlations between the epithelial alarmin levels in the serum and EBC of patients with IPF. Notes: Panels showing the correlation between: (**A**) IL-33 and TSLP serum levels, (**B**) IL-33 EBC levels and TSLP serum levels, (**C**) IL-33 serum levels and TSLP EBC levels, (**D**) IL-33 EBC levels and IL-33 serum levels. EBC—exhaled breath condensate IPF—idiopathic pulmonary fibrosis, IL-25—interleukin 25, IL-33—interleukin 33, TSLP—thymic stromal lymphopoietin.

We found a significant positive correlation between IL-33 and TSLP serum levels (*r* = 0.62, *p* < 0.0001). IL-33 EBC levels correlated negatively with TSLP serum levels (*r* = −0.37, *p* < 0.01). Similarly, IL-33 serum levels correlated negatively with TSLP EBC levels (*r* = −0.28, *p* < 0.05). We also found a significant negative correlation between IL-33 EBC levels and IL-33 serum levels (*r* = −0.29, *p* < 0.05). Analysis of all possible correlations of epithelial alarmin levels in the serum and EBC is shown in Table S1. The most relevant correlations are presented in Figure 3.

### 3.5. Associations between the Epithelial Alarmin Levels and Clinical Measures in Patients with IPF

**Figure 4:**
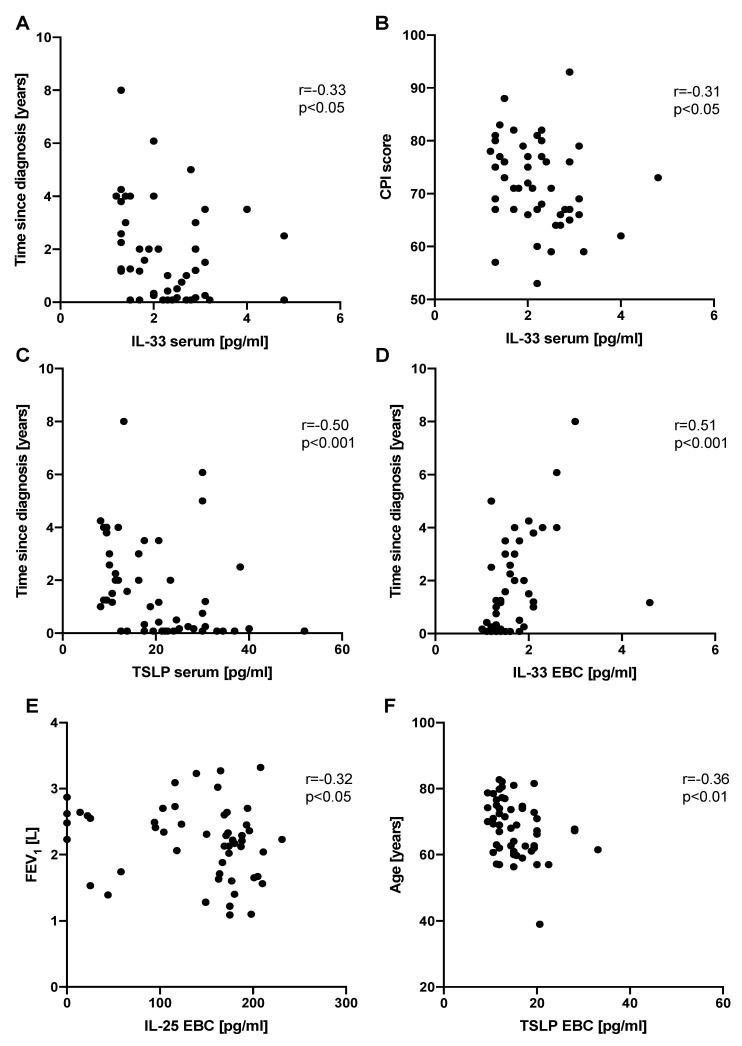
Graphical presentation of significant correlations between the epithelial alarmin levels and clinical measures in patients with IPF. Notes: Panels showing correlation between: (**A**) IL-33 serum levels and time since diagnosis, (**B**) IL-33 serum levels and CPI score, (**C**) TSLP serum levels and time since diagnosis, (**D**) IL-33 EBC levels and time since diagnosis, (**E**) IL-25 EBC levels and FEV_1_, (**F**) TSLP EBC levels and age. IPF—idiopathic pulmonary fibrosis, EBC—exhaled breath condensate, IL-25—interleukin 25, IL-33—interleukin 33, TSLP—thymic stromal lymphopoietin, FEV_1_—forced expiratory volume in 1 second, CPI—composite physiologic index.

IL-33 serum levels correlated negatively with time since diagnosis (*r* = −0.33, *p* < 0.05) as well as with CPI score (*r* = −0.31, *p* <.05). TSLP serum levels also correlated negatively with time since diagnosis (*r* = −0.50, *p* < 0.001). Significant negative correlations were observed between IL-25 EBC levels and FEV_1_ (*r* = −0.32, *p* < 0.05) as well as TSLP EBC levels and age (*r* = −0.36, *p* < 0.01). IL-33 EBC levels correlated positively with time since diagnosis (*r* = 0.51, *p* < 0.001). All possible correlations between epithelial alarmin levels and clinical measures in patients with IPF are presented in Table S2. The most relevant and significant correlations are shown in Figure 4.

### 3.6. Changes in the Epithelial Alarmin Levels in the Serum of Patients with IPF before and after 12 Months of Antifibrotic Therapy

Baseline and follow-up serum and EBC concentrations of epithelial alarmins were measured in 35 out of 52 IPF subjects studied. The summary of the characteristics of the follow-up cohort is shown in Table 2. IL-33 serum follow-up levels (2.00 (1.70–2.40) pg/mL) did not differ significantly from baseline levels (2.20 (1.40–2.90) pg/mL). Similarly, TSLP serum follow-up levels (15.63 (11.88–17.50) pg/mL) were not significantly different from baseline levels (16.25 (10.00–22.50) pg/mL), see Figure S1. IL-25 serum levels were measurable only in a few subjects studied; therefore, the analysis of changes was not performed.

Additional analysis was performed according to the antifibrotic agent used during 12 months of therapy. Among 35 IPF subjects in the follow-up cohort, 10 subjects were treated with nintedanib and 25 subjects were treated with pirfenidone. Among the subgroup of patients treated with nintedanib, IL-33 serum follow-up levels did not differ significantly from baseline levels (2.27 (1.66–2.39) pg/mL vs. 2.09 (1.41–2.88) pg/mL, respectively) as well as TSLP serum follow-up levels were not different from baseline levels (15.63 (11.25–16.88) pg/mL vs. 23.44 (10.63–30.00) pg/mL, respectively). Similarly, in the subgroup of patients treated with pirfenidone, IL-33 serum baseline levels did not differ significantly from follow-up levels (2.21 (1.47–2.82) pg/mL vs. 1.96 (1.66–2.33) pg/mL, respectively) as well as TSLP serum baseline levels were not different compared to follow-up levels (16.25 (10.00–22.50) pg/mL vs. 15.63 (11.88–17.50) pg/mL, respectively). Changes in IL-33 and TSLP serum levels of patients with IPF before and after 12 months of antifibrotic therapy according to the antifibrotic agent used are presented in Figure S2.

### 3.7. Changes in the Epithelial Alarmin Levels in the EBC before and after 12 Months of Antifibrotic Therapy

**Figure 5:**
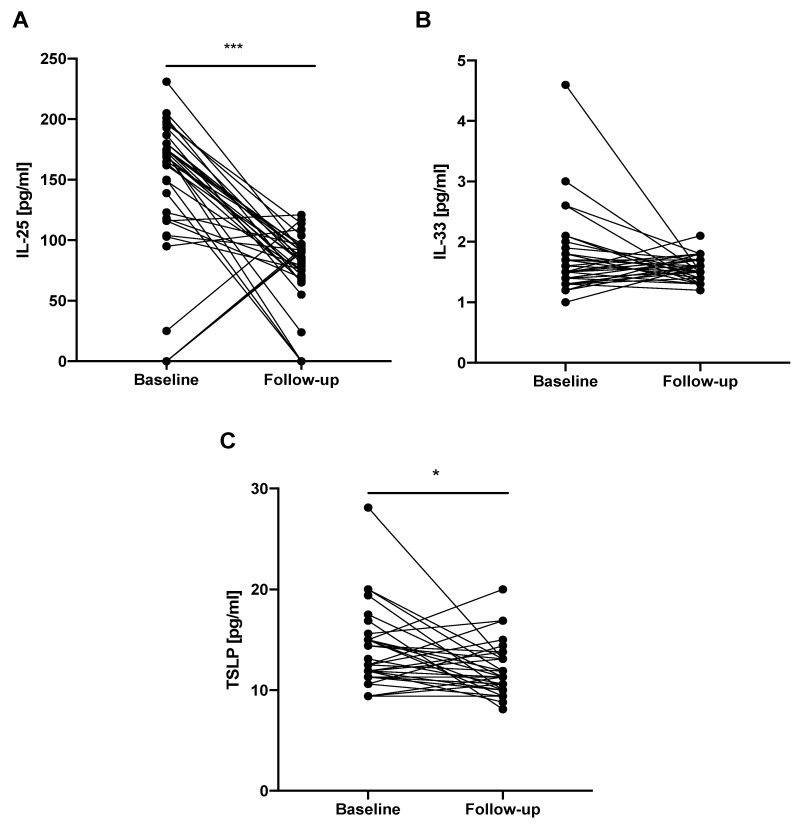
Changes in the epithelial alarmin levels in the EBC of patients with IPF before and after 12 months of antifibrotic therapy. Notes: Panels showing: (**A**) change in IL-25 EBC level; (**B**) change in IL-33 EBC level; (**C**) change in TSLP EBC level; * *p* < 0.05, *** *p* < 0.001. EBC—exhaled breath condensate, IPF—idiopathic pulmonary fibrosis, IL-25—interleukin 25, IL-33—interleukin 33, TSLP—thymic stromal lymphopoietin.

IL-33 EBC follow-up levels (1.47 (1.41–1.66) pg/mL) did not differ significantly from baseline levels (1.47 (1.35–1.84) pg/mL). We noted significantly decreased follow-up IL-25 EBC levels (84.47 (69.97–93.8) pg/mL) compared to baseline levels (168.8 (117.13–177.47) pg/ml; *p* < 0.0001). Similarly, TSLP EBC follow-up levels were significantly decreased (11.25 (10.0–13.13*) pg/mL) compared to baseline levels (12.5 (11.56–15.0) pg/mL; *p* < 0.05), see Figure 5.

**Figure 6:**
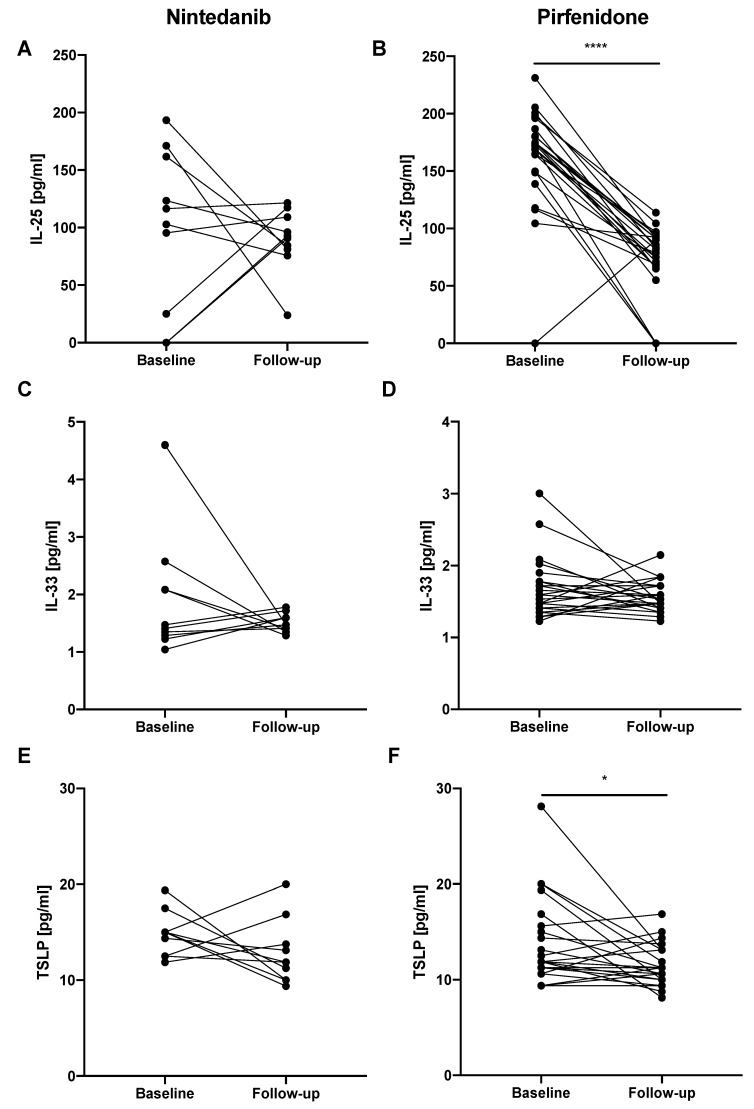
Changes in the epithelial alarmin levels in the EBC of patients with IPF before and after 12 months of antifibrotic therapy according to the antifibrotic agent used. Notes: Panels showing: (**A**) change in IL-25 EBC level in IPF patients treated with nintedanib; (**B**) change in IL-25 EBC level in IPF patients treated with pirfenidone; (**C**) change in IL-33 EBC level in IPF patients treated with nintedanib; (**D**) change in IL-33 EBC level in IPF patients treated with pirfenidone; (**E**) change in TSLP EBC level in IPF patients treated with nintedanib; (**F**) change in TSLP EBC level in IPF patients treated with pirfenidone; * *p* < 0.05, **** *p* < 0.0001. Abbreviations: EBC—exhaled breath condensate, IPF—idiopathic pulmonary fibrosis, IL-25—interleukin 25, IL-33—interleukin 33, TSLP—thymic stromal lymphopoietin.

In the next step, additional analysis was performed according to the antifibrotic agent used during the 12 months of therapy. In the subgroup of IPF patients treated with nintedanib, IL-33 EBC follow-up levels did not differ significantly from baseline levels (1.47 (1.41–1.60) pg/mL vs. 1.44 (1.29–2.09) pg/mL, respectively) as well as TSLP EBC follow-up levels were not different from baseline levels (11.88 (10.00–13.75) pg/mL vs. 15.00 (12.50–15.00) pg/mL, respectively). Similarly, IL-25 EBC baseline levels did not differ significantly from follow-up levels (109.63 (25.13–161.80) pg/mL vs. 91.80 (81.13–109.13) pg/mL, respectively). In the subgroup of IPF patients treated with pirfenidone we noted significantly decreased IL-25 EBC follow-up levels (81.80 (66.80–91.47) pg/mL) compared to baseline levels (173.13 (149.80-180.47) pg/mL; *p* < 0.0001). The same observation was noted for decreased TSLP EBC follow-up levels (11.25 (10.00–13.13) pg/mL) compared to baseline levels (11.88 (11.25–15.00) pg/mL; *p* < 0.05). IL-33 EBC baseline levels did not differ significantly from follow-up levels (1.53 (1.35–1.78) pg/mL vs. 1.53 (1.47–1.72) pg/mL, respectively). All changes of the epithelial alarmin levels in the EBC of patients with IPF before and after 12 months of antifibrotic therapy according to the antifibrotic agent used are presented in Figure 6.

### 3.8. Changes in the Epithelial Alarmin Levels in the Serum of Patients with IPF before and after 12 Months of Antifibrotic Therapy According to the Functional Decline

Additional analysis based on the annual rate of decline in absolute FVC % of predicted value was performed. IPF subjects were classified as stables (absolute FVC % of predicted decline/year ≤ 5%, *n* = 25) and progressors (absolute FVC % of predicted decline/year > 5%, *n* = 10). Table 3 summarizes the functional decline in the studied cohort of patients with IPF.

In the subgroup of stable IPF patients, IL-33 serum follow-up levels did not differ significantly from baseline levels (1.96 (1.66–2.36) pg/mL vs. 2.21 (1.38–2.73) pg/mL, respectively) as well as TSLP serum follow-up levels (16.25 (14.38–17.82) pg/mL) were not different from baseline levels (16.25 (10.32–23.75) pg/mL). Similarly, in the subgroup of progressors, IL-33 serum baseline levels did not differ significantly from follow-up levels (2.48 (1.49–3.00) pg/mL vs. 2.09 (1.63–2.41) pg/mL, respectively), as well as TSLP serum follow-up levels (11.25 (10.63–16.25) pg/ml) were not different from baseline levels (20.63 (9.69–32.03) pg/mL). Changes in IL-33 and TSLP serum levels of patients with IPF before and after 12 months of antifibrotic therapy according to the functional decline are presented in Figure S3.

### 3.9. Changes in the Epithelial Alarmin Levels in the EBC of Patients with IPF before and after 12 Months of Antifibrotic Therapy According to the Functional Decline

**Figure 7:**
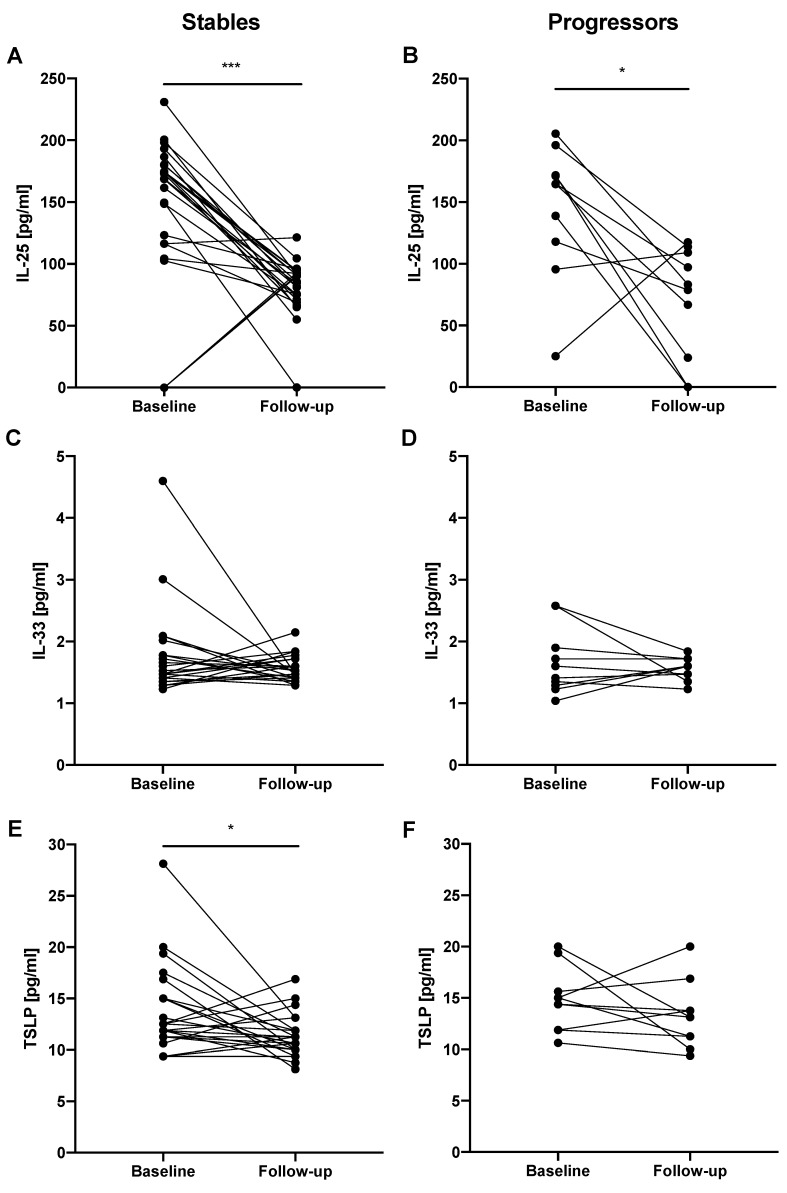
Changes in the epithelial alarmin levels in the EBC of patients with IPF before and after 12 months of antifibrotic therapy according to the functional decline (stables—FVC % of predicted decline/year ≤ 5%; progressors—FVC % of predicted decline/year > 5%). Notes: Panels showing: (**A**) change in IL-25 EBC levels in stable IPF patients; (**B**) change in IL-25 EBC levels in progressor IPF patients; (**C**) change in IL-33 EBC levels in stable IPF patients; (**D**) change in IL-33 EBC levels in progressor IPF patients; (**E**) change in TSLP EBC levels in stable IPF patients; (**F**) change in TSLP EBC levels in progressor IPF patients; * *p* < 0.05, *** *p* < 0.001. Abbreviations: EBC—exhaled breath condensate, IPF—idiopathic pulmonary fibrosis, IL-25—interleukin 25, IL-33—interleukin 33, TSLP—thymic stromal lymphopoietin.

In the subgroup of stable IPF patients, IL-33 EBC follow-up levels (1.47 (1.41–1.66) pg/mL) did not differ significantly from baseline levels (1.47 (1.35–1.90) pg/mL). We noted significantly decreased follow-up IL-25 EBC levels (85.80 (73.13−92.64) pg/mL) compared to baseline levels (169.1 (116.50–180.30) pg/mL; *p* < 0.0001). Similarly, TSLP EBC follow-up levels were significantly decreased (11.25 (10.0–11.88) pg/mL) compared to baseline levels (11.88 (11.25–110.30) pg/mL; *p* < 0.05). In the subgroup of progressors IL-33 EBC baseline levels did not differ significantly from follow-up levels (1.51 (1.28−2.07) pg/mL vs. 1.60 (1.44–1.72) pg/mL, respectively), as well as TSLP EBC follow-up levels were not different from baseline levels (13.13 (10.94–14.53) pg/mL vs. 14.69 (11.88–16.57) pg/mL, respectively). We noted significantly decreased IL-25 EBC follow-up levels (80.97 (17.85–92.64) pg/mL) compared to baseline levels (164.60 (112.20–177.90) pg/mL; *p* < 0.01). Changes in the epithelial alarmin levels in the EBC of patients with IPF before and after 12 months of antifibrotic therapy according to the functional decline are shown in Figure 7.

## 4. Discussion

In the present study, we aimed to assess prospectively epithelial alarmin concentrations in the systemic and lung compartment in the cohort of patients diagnosed with IPF and enrolled in antifibrotic therapy at our institution. In addition, we compared baseline alarmin levels in the serum and EBC obtained from IPF patients with control subjects and explored their possible associations with clinical characteristics. The main findings of this study are that TSLP serum and EBC concentrations are elevated in patients with IPF compared to controls. Neither IL-25 nor IL-33 serum or EBC levels differ significantly between IPF patients and control subjects. We demonstrate that IL-25 and TSLP EBC levels significantly decrease during antifibrotic treatment, which is mainly driven by the use of pirfenidone as an antifibrotic agent. We also show that IL-25 and TSLP EBC levels significantly decrease over a study period in stable patients with IPF, whereas in progressors a significant decrease is noted only in IL-25 EBC levels. Taken together, these findings confirm that epithelial alarmins play significant roles in the pathobiology of IPF and antifibrotic agents might affect their expression in the lung compartment.

The airway and alveolar epithelium functions as an essential physical and immunological barrier that responds to the environmental stimuli as a critical immune regulator through the secretion of cytokines, chemokines, growth factors, antimicrobial peptides, and the recruitment of leukocytes. The respiratory epithelium is ideally situated to orchestrate and impact respiratory system adaptive immune responses, and functions as an important interface between innate and adaptive immune regulation mechanisms. In the above context, epithelial production of alarmins, including IL-25, IL-33, and TSLP has emerged as critical epithelial factors that can initiate and/or amplify dysregulated immune responses in respiratory diseases [35]. These three epithelial-derived cytokines are important initiators of type 2 immunity, and their expression during type 2 related disease in humans is well documented [1,2,3,36,37,38]. Their biological role focuses mainly on triggering the production of the type 2 cytokines, including IL-5, IL-9, and IL-13 by diverse effector cells of the innate and adaptive immune system [4,5]. It is worthy of note that IL-13 is an important inducer of fibrosis in several chronic lung diseases, including IPF [39].

Although traditionally associated with allergic inflammation, type 2 responses are also recognized to contribute to the pathogenesis of non-allergen driven immune responses leading to tissue fibrosis [40,41]. However, the role of epithelial alarmins in the development of non-allergen driven respiratory diseases, characterized by profibrotic type 2 immune phenotypes is underexplored.

Recently studies employing animal models proved that IL-25, IL-33, and TSLP have an emergent role in the generation of pulmonary fibrosis [6,7,9,10,13,14,18]. Moreover, all three alarmins have been found at significantly higher levels than in healthy controls in the lung cell cultures or bronchoalveolar lavage (BAL) fluid of patients with IPF [6,8,9,10,15,42]. However, the precise roles of epithelial alarmins in the pathogenesis of IPF remain poorly understood.

Our study aimed to analyze the epithelial alarmin levels in the systemic and local lung compartments. We noted only TSLP serum and EBC levels to be significantly overexpressed compared with control subjects. It has been shown previously that TSLP and its receptor are highly upregulated in the lungs of patients with IPF and that this mediator is an important regulator of type 2 immune responses in chronic fibrotic lung disease [15]. We failed to confirm our previous findings of elevated IL-33 EBC levels in patients with IPF compared to healthy subjects [26]. In this study, both systemic and lung compartment expression of IL-33 and IL-25 were not different between patients with IPF and controls. It should be noted that IL-33 was found to promote profibrotic immune responses triggered by various insults in IPF [6,7,13,14]. Similarly, it has recently been shown that IL-25 is upregulated in IPF lung tissue samples and promotes lung fibrosis by directly mediating alveolar epithelial cells and fibroblast activation [10]. Noted discrepancies between our results and others reporting elevated levels of all three alarmins in the lung cell cultures or BAL fluid samples of patients with IPF [6,8,9,10,15,42] might be explained by different expression of the epithelial alarmins in the serum and EBC compared to lung tissue or BAL fluid.

Analysis of possible associations between alarmins in the systemic and lung compartments showed a moderate positive association between TSLP and IL-33 serum levels, and a weak negative association between TSLP serum and IL-33 EBC levels, TSLP EBC and IL-33 serum levels, and IL-33 EBC and IL-33 serum levels. Lack of uniform association between alarmins in the serum and EBC may be a consequence of different biologic expression of the epithelial alarmins in the systemic and local lung compartments and overlapping functional activities of these cytokines [43,44].

Further analysis in our study focused on the associations between the epithelial alarmins and clinical measures in patients with IPF. Only TSLP EBC levels had a weak negative correlation with the age of IPF subjects. When analyzing associations between the epithelial alarmins and lung function parameters, we found a weak negative association of IL-25 serum levels and FEV_1_. We failed to confirm our previous finding of a negative association of IL-33 EBC concentrations with T_LCO_ [26]. Although, we noted a weak negative association of circulating IL-33 with disease severity expressed with the composite physiologic index (CPI). CPI reflects the morphologic extent of pulmonary fibrosis more accurately than individual lung function indices, provides prognostic information in patients with IPF, and may be used for staging the disease for clinical practice [33]. We also found weak negative associations between disease duration (time since diagnosis) and both IL-33 and TSLP serum levels, which has not been reported previously in a study of the epithelial alarmins expression in IPF [8]. Taken together, we hypothesize that one possible explanation for the above findings could be a greater release of epithelial alarmins in the lung compartment with subsequent systemic absorption at early stages of initiation of the pathogenic process in IPF, and decreased release of these cytokines when the pathogenic process is chronically perpetuated, lung fibrosis is more advanced, and scar tissue replaces extensively normal epithelial cells, which leads finally to progressive lung function impairment. However, we have found a moderate positive association between IL-33 EBC levels and disease duration, which is a surprisingly contradictory result to our previous finding of a negative association between these two parameters [26] and stands opposite to the generated hypothesis. Thus, further studies of the epithelial alarmins expression in patients with IPF are warranted to clarify conflicting results and verify the above working hypothesis.

To the best of the authors’ knowledge, no previous studies on epithelial alarmins in patients with IPF analyzed their concentrations in the systemic and lung compartment prospectively. In the present study, we have shown that both IL-25 and TSLP expression in patients with IPF decreases significantly in the lung compartment after 12 months of antifibrotic treatment. Systemic compartment levels did not change over that time. Interestingly, we demonstrated that significant changes in IL-25 and TSLP EBC levels before and after 12 months of antifibrotic therapy were observed in the subgroup of IPF patients treated with pirfenidone, but not in the subgroup of patients treated with nintedanib. Both nintedanib and pirfenidone, independently, have been shown to slow the disease progression limiting the decline of lung function in patients with IPF [45,46]. Both medications are recognized as an actual standard of pharmacological treatment of the disease [47]. However, their mechanisms of action vary significantly. Nintedanib has been shown to inhibit receptor tyrosine kinase signaling by the platelet-derived growth factor, fibroblast growth factor, and vascular endothelial growth factor [48]. The mode of action of pirfenidone seems to be more pleiotropic and has not been fully recognized. Pirfenidone has been shown to have antifibrotic, anti-inflammatory, and antioxidative properties, although inhibition of transforming growth factor β (TGF-β) is often suggested as the most important [49,50,51,52,53]. We, therefore, speculate that pirfenidone, due to more complex and not fully known actions, may exert pharmacodynamic effects on the lung compartment levels of epithelial alarmins. As far as we know, no previous studies reported that IL-25 and TSLP pathways leading to lung fibrosis might be impacted by pirfenidone.

Moreover, our study shows that stable patients with IPF (18 on pirfenidone and 7 on nintedanib) had a significant decrease in the EBC levels of both IL-25 and TSLP after one year of antifibrotic therapy. Whereas, progressors (7 on pirfenidone and 3 on nintedanib) during the study period had a significant decrease in IL-25 EBC levels only. Interestingly, it has been shown that due to overlapping and redundant roles of IL-25, IL-33, and TSLP in the maintenance of type 2 responses, only combined targeting of these cytokines (more than one at a time) ameliorates progressive type-2 driven inflammation and fibrosis [44]. We and others, therefore, proved that only combined suppression of epithelial alarmins might translate to improved outcomes in type 2 chronic respiratory conditions.

Recent work has highlighted a novel and important finding of the constitutive expression of a functional TSLP receptor (TSLPR) complex on fibroblasts and a significant role of TSLP-TSLPR signaling axis in type 2 responses and organ fibrosis [15]. Based on our study finding of elevated systemic and lung compartment levels of TSLP in patients with IPF compared to controls and a finding of a significant decrease of TSLP lung compartment levels after 12 months of antifibrotic therapy observed in stable patients with IPF, but not in progressors, we speculate, that targeting TSLP functional signaling axis in a fibrotic lung disease may have a therapeutic potential in fibroproliferative lung diseases, such as IPF.

These novel exploratory findings must be considered in the context of the present study limitations. Among several limitations, the most important are relatively small sample size and the difference in age and smoking history between IPF subjects and controls. The difference in the number of patients treated with each of the antifibrotics in the present study resulted from earlier drug reimbursement and availability of pirfenidone in our country. It is of note that such a difference could affect the obtained results of prospective changes in epithelial alarmin concentrations in the present study. However, this study was exploratory and not confirmatory; therefore, the sample size estimation and power analysis were not calculated. Cigarette smoke is a well-recognized trigger for the epithelial production of alarmins; thus, the difference in smoking exposure could affect the results. Given that only TSLP EBC levels correlated weakly with the age of IPF patients, there is a possibility that our study baseline results were not affected by the difference in age between studied cohorts. Furthermore, the lack of the comparative group of patients with IPF not treated with antifibrotics is a significant drawback of the prospective analysis of the epithelial alarmin levels in the present study. Although, it would be unethical to enroll IPF subjects into the prospective follow-up study and not to offer them an effective treatment. Despite the above limitations, in our view, the novelty of findings extends our current knowledge on the importance of epithelial alarmins in the pathobiology of IPF.

## 5. Conclusions

The findings of this study strengthen the shreds of evidence that the epithelial alarmins play significant roles in the pathobiology of IPF. Elevated systemic and lung compartment TSLP levels in patients with IPF compared to controls and their significant decrease in the lung compartment during antifibrotic therapy in stable patients with IPF, but not in progressors, support its significant contribution to profibrotic type 2 immune responses in IPF. Noted changes in the epithelial alarmin concentrations in the lung compartment during pirfenidone therapy may suggest its possible impact on the immunologic responses in IPF driven by IL-25 and TSLP. Certainly, further studies in larger IPF cohorts, including studies analyzing the possible mechanistic link between antifibrotics actions and the epithelial alarmins expression are required to validate present study findings and generated hypotheses.

## Figures and Tables

**Table 1:** Characteristics of study participants.

	Controls	IPF
**Number of subjects**	30	52
Sex (male/female)	17/13	39/13
Age (years), mean (SD)	61.36 (7.26)	68.21 (8.76) ***
Smoking exposure (pack-years) median (IQR)	8 (0–25)	26.5 (7.75–39) **
Smoking status		
never smokers, *n* (%)	12 (40.00)	12 (23.07)
ex-smokers, *n* (%)	8 (26.67)	37 (71.15)
current smokers, *n* (%)	10 (33.33)	3 (5.77)
GAP score, median (IQR)	N/A	4.0 (3.0–4.5)
GAP index:	N/A	
Stage I, *n* (%)		24 (46.15)
Stage II, *n* (%)		27 (51.92)
Stage III, *n* (%)		1 (1.92)
CPI score, mean (SD)	N/A	71.42 (8.41)
Time since diagnosis (years), median (IQR)	N/A	1.17 (0.08–2.79)
FEV_1_ (l), mean (SD)	3.07 (0.81)	2.21 (0.56) ****
FEV_1_ (% of predicted), mean (SD)	100.88 (14.59)	77.16 (17.08) ****
FVC (l), mean (SD)	4.09 (1.10)	2.74 (0.78) ****
FVC (% of predicted), mean (SD)	103.97 (14.74)	72.96 (16.87) ****
FEV_1_/FVC%, mean (SD)	75.26 (4.83)	81.67 (7.61) ****
T_LCO_ (mmol/min/kPa), median (IQR)	N/A	3.7 (2.97–4.59)
T_LCO_ (% of predicted), mean (SD)	N/A	49.29 (13.61)
6MWT (meters), mean (SD)	N/A	378.37 (113.31)

Notes: ** *p* < 0.01; *** *p* < 0.001; **** *p* < 0.0001. IPF—idiopathic pulmonary fibrosis, FEV_1_—forced expiratory volume in 1 second, FVC—forced vital capacity, T_LCO_—transfer factor of the lung for carbon mon oxide, 6MWT—six-minute walk test, GAP—gender, age, and 2 physiology variables (FVC and T_LCO_), CPI—composite physiologic index, N/A—not applicable.

**Table 2:** Characteristics of the IPF follow-up cohort.

	Baseline		12-Months Follow-Up	
**Number of subjects**	35		35	
Sex (male/female)	24/11		24/11	
Age (years), mean (SD)	68.78 (7.81)		69.78 (7.81)	
Smoking exposure (pack-years) median (IQR)	28.00 (0–40.00)		28.00 (0–40.00)	
Smoking status				
never smokers, *n* (%)	8 (22.86)		8 (22.86)	
ex-smokers, *n* (%)	25 (71.43)		25 (71.43)	
current smokers, *n* (%)	2 (5.71)		2 (5.71)	
GAP score, median (IQR)	3.00 (3.00–4.00)		4.00 (3.00–5.00) ***	
GAP index:				
Stage I, *n* (%)	21 (60.00)		10 (28.57)	
Stage II, *n* (%)	14 (40.00)		23 (65.71)	
Stage III, *n* (%)	0 (0.00)		2 (5.71)	
CPI score, mean (SD)	69.69 (7.90)		74.97 (8.67) ****	
Time since diagnosis (years), median (IQR)	1.25 (0.17–3.50)		2.25 (1.17–4.50) ****	
FEV_1_ (l), mean (SD)	2.20 (0.58)		2.11 (0.56) *	
FEV_1_ (% of predicted), mean (SD)	78.84 (18.36)		77.04 (19.50)	
FVC (l), mean (SD)	2.75 (0.82)		2.64 (0.78) *	
FVC (% of predicted), mean (SD)	74.85 (17.93)		72.98 (18.62)	
FEV_1_/FVC%, mean (SD)	81.25 (7.42)		81.13 (8.03)	
T_LCO_ (mmol/min/kPa), mean (SD)	4.04 (1.11)		3.36 (1.18) ****	
T_LCO_ (% of predicted), mean (SD)	52.58 (12.99)		43.86 (15.28) ****	
6MWT (meters), mean (SD)	389.00 (106.86)		368.00 (125.22)	
	Nintedanib	Pirfenidone	Nintedanib	Pirfenidone
Number of subjects	10	25	10	25
Sex (male/female)	7/3	17/8	7/3	17/8
Age (years), mean (SD)	68.42 (8.64)	68.92 (7.63)	69.42 (8.64)	69.92 (7.63)
Smoking exposure (pack-years), median (IQR)	37.70 (0.00–46.00)	25.00 (5.50–30.00)	37.70 (0.00–46.00)	25.00 (5.50–30.00)
Smoking status				
never smokers, *n* (%)	3 (30.00%)	5 (20.00)	3 (30.00)	5 (20.00)
ex-smokers, *n* (%)	6 (60.00%)	19 (76.00)	6 (60.00)	19 (76.00)
current smokers, *n* (%)	1 (10.00%)	1 (4.00)	1 (10.00)	1 (4.00)
GAP score, median (IQR)	3.00 (2.00–3.00)	3.00 (3.00–4.00)	4.00 (3.00–5.00) ***	4.00 (3.00–5.00) **
GAP index:				
Stage I, *n* (%)	8 (80.00)	13 (52.00%)	3 (30.00)	7 (28.00)
Stage II, *n* (%)	2 (20.00)	12 (48.00%)	7 (70.00)	16 (64.00)
Stage III, *n* (%)	0 (0.00)	0 (0.00)	0 (0.00)	2 (8.00)
CPI score, mean (SD)	71.20 (6.94)	69.08 (8.31)	78.20 (6.94) ***	73.68 (9.07) ***
Time since diagnosis (years), median (IQR)	1.19 (0.17–3.00)	1.58 (0.33–3.50)	2.19 (1.17–4.00) **	2.58 (1.33–4.50) ****
FEV_1_ (l), mean (SD)	2.47 (0.44)	2.10 (0.60)	2.35 (0.57)	2.02 (0.54)
FEV_1_ (% of predicted), mean (SD)	87.00 (15.58)	75.57 (18.64)	84.01 (21.01)	74.26 (18.58)
FVC (l), mean (SD)	3.09 (0.70)	2.62 (0.84)	2.92 (0.82)	2.53 (0.75)
FVC (% of predicted), mean (SD)	83.09 (17.02)	71.55 (17.53)	79.33 (21.11)	70.43 (17.33)
FEV_1_/FVC%, mean (SD)	80.95 (8.36)	81.37 (7.20)	81.43 (9.99)	81.01 (7.34)
T_LCO_ (mmol/min/kPa), mean (SD)	4.20 (1.19)	3.98 (1.09)	3.24 (1.14) ***	3.40 (1.21) ****
T_LCO_ (% of predicted), mean (SD)	52.68 (10.59)	52.54 (14.04)	40.43 (12.58) ***	45.23 (16.26) ***
6MWT (meters), mean (SD)	388.20 (72.61)	389.32 (119.16)	368.89 (56.74)	367.68 (143.13)

Notes: * *p* < 0.05; ** *p* < 0.01; *** *p* < 0.001; **** *p* < 0.0001. IPF—idiopathic pulmonary fibrosis, FEV_1_—forced expiratory volume in 1 second, FVC—forced vital capacity, T_LCO_—transfer factor of the lung for carbon monoxide, GAP—gender, age, and 2 physiology variables (FVC and T_LCO_), CPI—composite physiologic index, 6MWT—six-minute walk test.

**Table 3:** Functional decline in the studied cohort of patients with IPF.

	Entire IPF Follow-Up Cohort	Stables	Progressors
Number of subjects	35	25	10
FVC decline per year (l), median (IQR)	0.09 (−0.04–0.21)	0.01 (−0.11–0.12)	0.31 (0.20–0.54) ****
FVC decline per year (% of predicted), median (IQR)	2.01 (−2.10–6.11)	−0.69 (−3.35–2.18)	6.98 (6.28–13.12) ****

Notes: Negative values mean improvement of FVC, **** *p* < 0.0001. IPF—idiopathic pulmonary fibrosis, FVC—forced vital capaciy.

## Supplementary Materials

The following are available online at https://www.mdpi.com/2077-0383/8/10/1590/s1, Figure S1. Changes in the epithelial alarmin levels in the serum of patients with IPF before and after 12 months of antifibrotic therapy; Figure S2. Changes in the epithelial alarmin levels in the serum of patients with IPF before and after 12 months of antifibrotic therapy according to the antifibrotic agent used; Figure S3. Changes in the epithelial alarmin levels in the serum of patients with IPF before and after 12 months of antifibrotic therapy according to the functional decline (stables—FVC % of predicted decline/year ≤ 5%; progressors—FVC % of predicted decline/year > 5%); Table S1. Correlations of the epithelial alarmin levels in the serum and EBC of patients with IPF; Table S2. Correlations between the epithelial alarmin levels and clinical measures in patients with IPF.
