# Supplementary material for: Epithelial Alarmins in Serum and Exhaled Breath in Patients with Idiopathic Pulmonary Fibrosis: A Prospective One-Year Follow-Up Cohort Study
Source: J Clin Med. 2019 Oct 2;8(10):1590. doi: 10.3390/jcm8101590 (PMC6832270; doi:10.3390/jcm8101590)
Supplement: Supplementary file 1 [file jcm-08-01590-s001.pdf]

Journal of
Clinical Medicine

MDPI

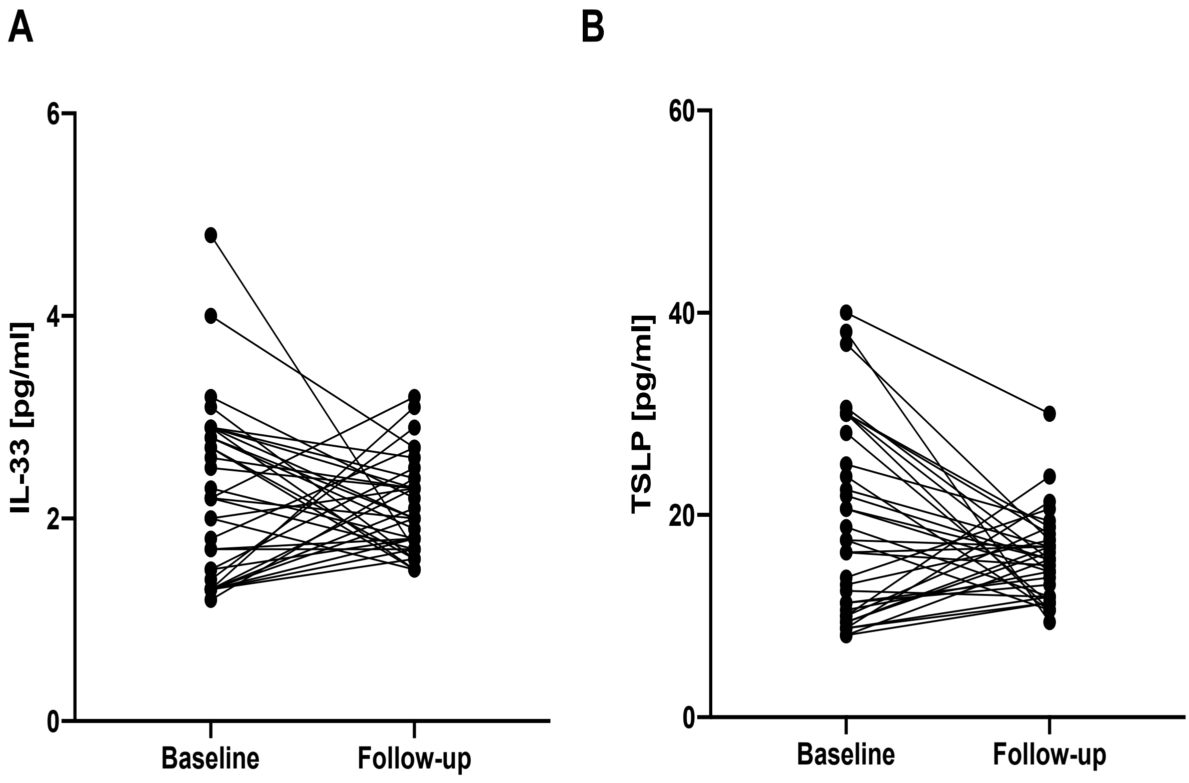

Figure S1. Changes in the epithelial alarmin levels in the serum of patients with IPF before and after 12 months of antifibrotic therapy

Notes: Panels showing: (**A**) change in IL-33 serum level; (**B**) change in TSLP serum level.

Abbreviations: IPF – idiopathic pulmonary fibrosis, IL-33 – interleukin 33, TSLP – thymic stromal lymphopoietin.

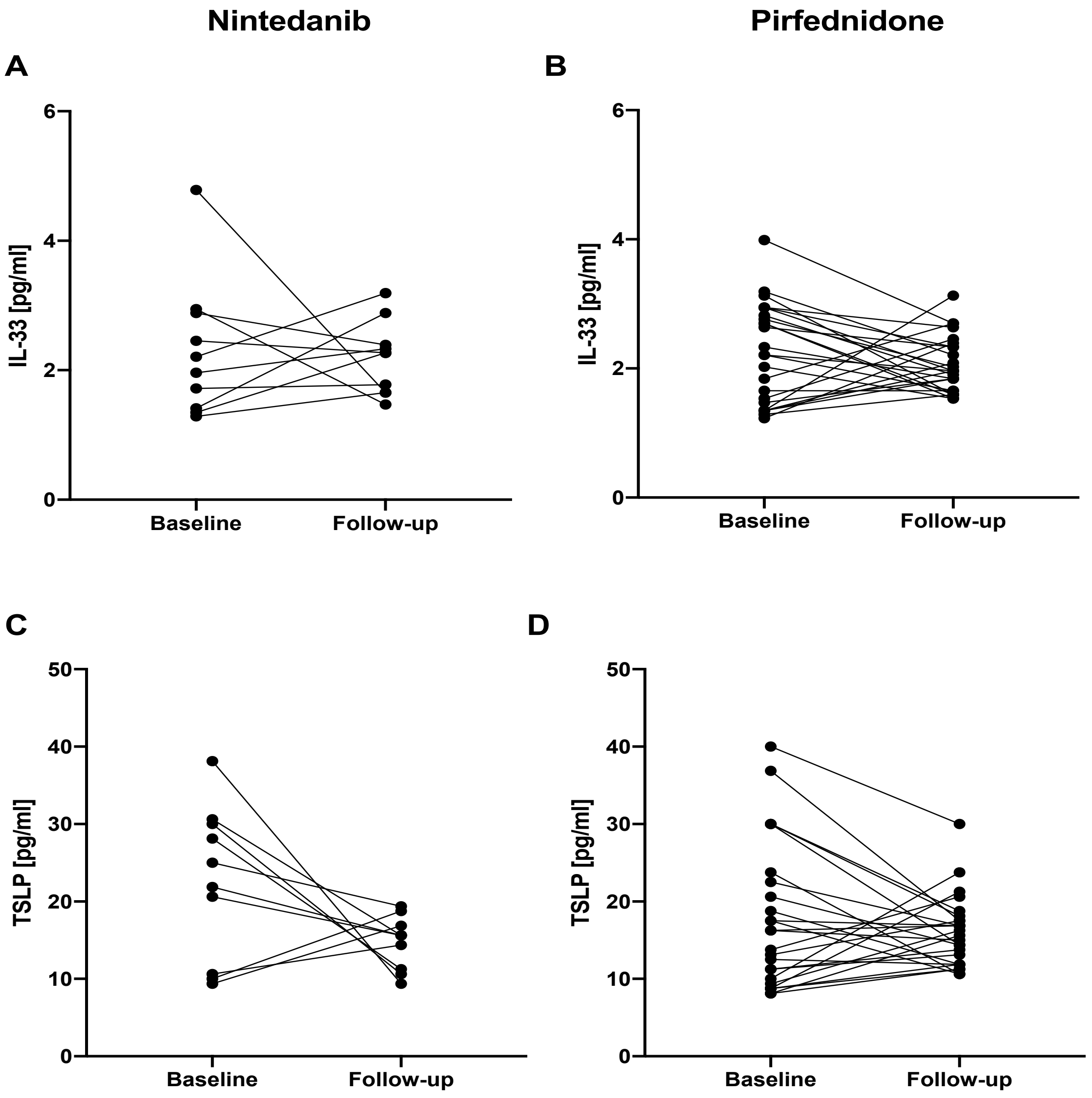

Figure S2. Changes in the epithelial alarmin levels in the serum of patients with IPF before and after 12 months of antifibrotic therapy according to the antifibrotic agent used.

Notes: Panels showing: (**A**) change in IL-33 serum level in IPF patients treated with nintedanib; (**B**) change in IL-33 serum level in IPF patients treated with pirfenidone; (**C**) change in TSLP serum level in IPF patients treated with nintedanib; (**D**) change in TSLP serum level in IPF patients treated with pirfenidone.

Abbreviations: IPF – idiopathic pulmonary fibrosis; IL-33 – interleukin 33, TSLP – thymic stromal lymphopoietin.

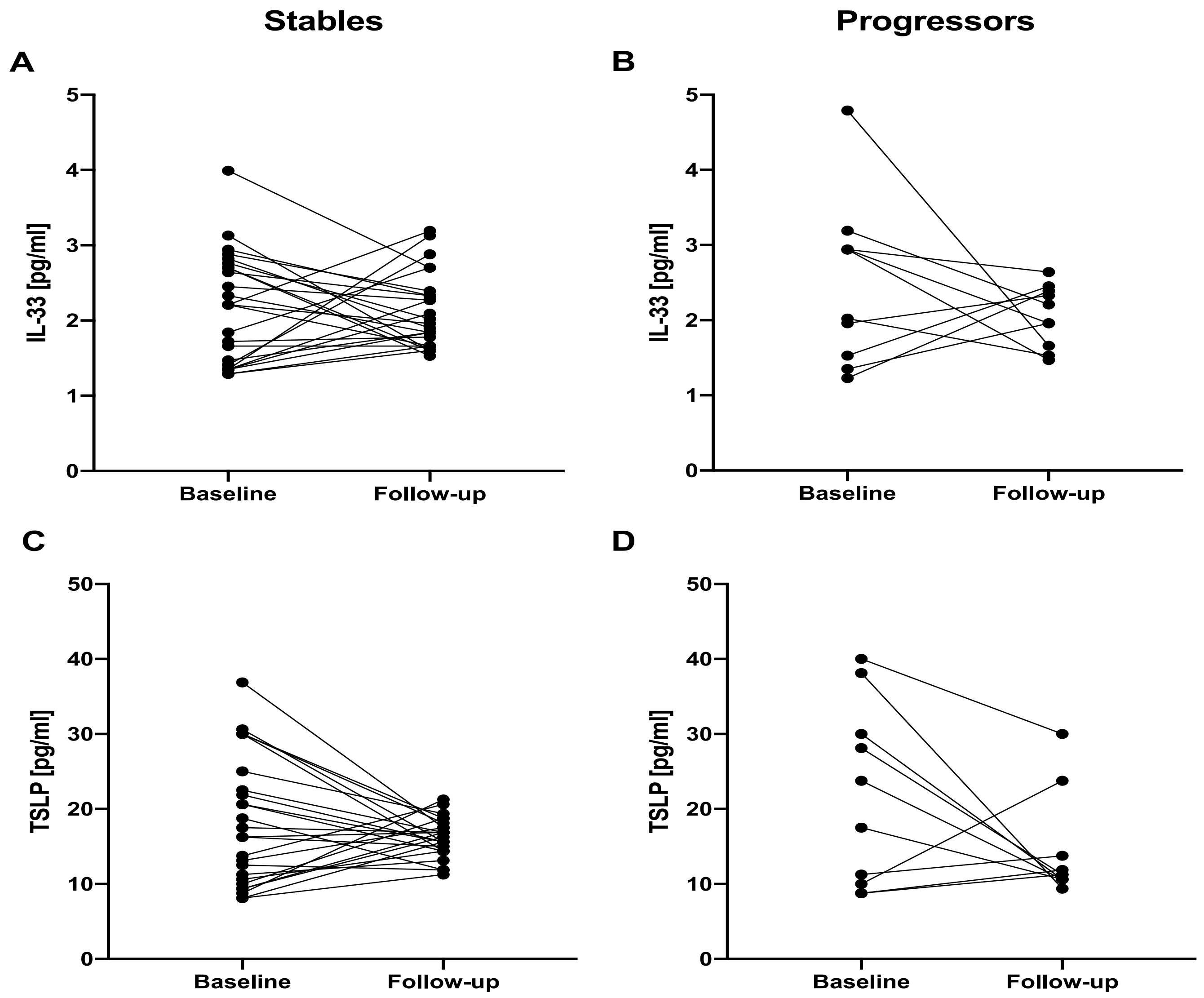

Figure S3. Changes in the epithelial alarmin levels in the serum of patients with IPF before and after 12 months of antifibrotic therapy according to the functional decline (stables – FVC % of predicted decline/year ≤ 5%; progressors – FVC % of predicted decline/year > 5%)

Notes: Panels showing: (**A**) change in IL-33 serum levels in stable IPF patients; (B) change in IL-33 serum levels in progressor IPF patients; (**C**) change in TSLP serum levels in stable IPF patients; (**D**) change in TSLP serum levels in progressor IPF patients.

Abbreviations: IPF – idiopathic pulmonary fibrosis; IL-33 – interleukin 33, TSLP – thymic stromal lymphopoietin.

Table S1. Correlations of the epithelial alarmin levels in the serum and EBC of patients with IPF.

		Serum			EBC		
		IL-33 (pg/ml)	IL-25 (pg/ml)	TSLP (pg/ml)	IL-33 (pg/ml)	IL-25 (pg/ml)	TSLP (pg/ml)
**Serum**	IL-33 (pg/ml)		r=0.12	**r=0.62********	**r=-0.29***	r=0.12	**r=-0.28***
	IL-25 (pg/ml)	r=0.12		**r=0.28***	r=-0.02	r=0.05	r=0.27
	TSLP (pg/ml)	**r=0.62********	**r=0.28***		**r=-0.37****	r=-0.03	r=-0.04
**EBC**	IL-33 (pg/ml)	**r=-0.29***	r=-0.02	**r=-0.37****		r=-0.17	r=0.05
	IL-25 (pg/ml)	r=0.12	r=0.05	r=-0.03	r=-0.17		r=-0.13
	TSLP (pg/ml)	**r=-0.28***	r=0.27	r=-0.04	r=0.05	r=-0.13	

Notes: *p<0.05; **p<0.01; **** p<0.0001
Abbreviations: EBC – exhaled breath condensate, IPF – idiopathic pulmonary fibrosis, IL-25 – interleukin 25, IL-33 – interleukin 33, TSLP – thymic stromal lymphopoietin.

Journal of Clinical Medicine MDPI

Table S2. Correlations between the epithelial alarmin levels and clinical measures in patients with IPF.

		FEV_1 (l)	FEV_1%	FVC (l)	FVC%	FEV_1/FVC%	T_{LCO} (mmol/min /kPa)	T_{LCO} % of predicted	Age (years)	Time since diagnosis (years)	Pack-years	GAP score	CPI score	6MWT (meters)
**Serum**	IL-33 (pg/ml)	r=-0.05	r=0.0007	r=-0.05	r=-0.03	r=0.15	r=0.14	r=0.20	r=-0.003	**r=-0.33***	r=0.04	r=-0.20	**r=-0.31***	r=-0.14
	IL-25 (pg/ml)	r=-0.10	**r=-0.30***	r=-0.02	r=-0.20	r=-0.11	r=-0.02	r=-0.05	r=-0.06	r=-0.12	r=-0.05	r=0.08	R=-0.04	r=-0.18
	TSLP (pg/ml)	r=0.02	r=0.003	r=0.07	r=0.04	r=-0.14	r=0.19	r=0.20	r=0.03	**r=-0.50*****	r=0.09	r=-0.14	R=-0.25	r=-0.17
**EBC**	IL-33 (pg/ml)	r=-0.01	r=0.05	r=-0.02	r=0.03	r=0.04	r=0.01	r=-0.0002	r=0.12	**r=0.51*****	r=-0.03	r=0.03	r=0.002	r=0.04
	IL-25 (pg/ml)	**r=-0.32***	r=-0.18	r=-0.25	r=-0.16	r=0.10	r=-0.08	r=0.006	r=-0.06	r=0.04	r=-0.02	r=-0.10	r=-0.06	r=0.006
	TSLP (pg/ml)	r=0.22	r=-0.05	r=0.23	r=-0.007	r=-0.06	r=-0.05	r=-0.23	**r=-0.36****	r=-0.08	r=0.04	r=0.05	r=0.25	r=0.12

Notes: * $p<0.05$; ** $p<0.01$; *** $p<0.001$.

Abbreviations: EBC – exhaled breath condensate, IPF – idiopathic pulmonary fibrosis, FEV_1 – forced expiratory volume in 1 second, FVC - forced vital capacity, T_{LCO} – transfer factor of the lung for carbon monoxide, GAP – gender, age, and 2 physiology variables (FVC and T_{LCO}), CPI – composite physiologic index, 6MWT – six-minute walk test.

© 2019 by the authors. Licensee MDPI, Basel, Switzerland. This article is an open access article distributed under the terms and conditions of the Creative Commons Attribution (CC BY) license (http://creativecommons.org/licenses/by/4.0/).
